# Circulating Levels of Dimethylarginines, Chronic Kidney Disease and Long-Term Clinical Outcome in Non-ST-Elevation Myocardial Infarction

**DOI:** 10.1371/journal.pone.0048499

**Published:** 2012-11-19

**Authors:** Viviana Cavalca, Fabrizio Veglia, Isabella Squellerio, Monica De Metrio, Mara Rubino, Benedetta Porro, Marco Moltrasio, Elena Tremoli, Giancarlo Marenzi

**Affiliations:** 1 Centro Cardiologico Monzino, I.R.C.C.S, Milan, Italy; 2 Dipartimento di Scienze Cliniche e di Comunità, Università degli Studi di Milano, Milan, Italy; 3 Dipartimento di Scienze Farmacologiche e Biomolecolari, Università degli Studi di Milano, Milan, Italy; S.G.Battista Hospital, Italy

## Abstract

**Background:**

Mechanisms linking chronic kidney disease (CKD) and adverse outcomes in acute coronary syndromes (ACS) are not fully understood. Among potential key players, reduced nitric oxide (NO) synthesis due to its endogenous inhibitors, asymmetric (ADMA) and symmetric (SDMA) dimethylarginine could be involved. We measured plasma concentration of arginine, ADMA and SDMA and investigated their relationship with CKD and long-term outcome in non-ST-elevation myocardial infarction (NSTEMI).

**Methodology/Principal Findings:**

We prospectively measured arginine, ADMA, and SDMA at hospital admission in 104 NSTEMI patients. CKD was defined as an estimated glomerular filtration rate (eGFR) <60 ml/min/1.73 m^2^. We considered a primary end point of combined cardiac death and re-infarction at a median follow-up of 21 months. In CKD (n = 33) and no-CKD (n = 71) patients, arginine and ADMA were similar, whereas SDMA was significantly higher in CKD patients (0.65±0.23 vs. 0.42±0.12 µmol/L; P<0.0001). Twenty-four (23%) patients had an adverse cardiac event during follow-up: 12 (36%) were CKD and 12 (17%) no-CKD patients (P = 0.02). When study population was stratified according to arginine, ADMA and SDMA median values, only SDMA (median 0.46 µmol/L) was associated with the primary end-point (P = 0.0016). In models adjusted for age, hemoglobin and left ventricular ejection fraction, the hazard ratio (HR) for CKD and SDMA were high (HR 2.93, interquartile range [IQR] 1.15–7.53; P = 0.02 and HR 6.80, IQR 2.09–22.2; P = 0.001, respectively) but, after mutual adjustment, only SDMA remained significantly associated with the primary end point (HR 5.73, IQR 1.55–21.2; P = 0.009).

**Conclusions/Significance:**

In NSTEMI patients, elevated SDMA plasma levels are associated with CKD and worse long-term prognosis.

## Introduction

Growing evidence suggests that chronic kidney disease (CKD) is associated with increased cardiovascular risk. Indeed, patients with CKD have both traditional and non-traditional (related to the underlying uremic state) risk factors, and the combination of these favors the development of cardiovascular disease, contributes to progression of CKD, and, ultimately, perpetuates mortality risk [1], [2]. The pervasive adverse influence of CKD has also been demonstrated in the setting of acute coronary syndromes (ACS). Among ACS patients, CKD doubles mortality rates and is third to cardiogenic shock and congestive heart failure as a predictor of mortality [3]–[7]. The mechanisms linking CKD and adverse outcomes in patients with ACS, however, are not fully understood. It is conceivable that the interplay among extensive co-morbidities, underutilization of known cardio-protective therapies, more frequent errors in dosing with excess toxicity from conventional therapies may explain part of their excessive risk, but other unidentified aspects related to the unique pathobiology of CKD are possibly involved [1], [7]–[9]. Recognition of the mechanisms associated with increased risk of ACS patients with CKD is a critical challenge to better determine where to concentrate the efforts in future trials and to validate novel and effective therapies for these high-risk patients.

Among other potential key players contributing to the increased cardiovascular risk of CKD patients, dimethylarginines have recently received attention. Reduced nitric oxide (NO) synthesis due to increased levels of its circulating endogenous inhibitor, asymmetric dimethylarginine (ADMA), has been suggested to independently contribute to progression of CKD, to end-stage renal disease, and death [10], [11]. Plasma concentrations of ADMA and of its isomer symmetric dimethylarginine (SDMA) are elevated in patients with CKD and in other cardiovascular risk-states such as hypertension, hypercholesterolemia, diabetes mellitus and chronic heart failure, and are associated with oxidative stress and endothelial dysfunction [12]–[18].

The role of these inhibitors of NO, as well as their relative levels in plasma, in ACS patients with CKD, are still unknown. Indeed, in the systemic, as well as in the coronary circulation, NO relaxes vascular smooth muscle to increase blood flow, and suppresses processes involved in vascular disease, including leukocyte adhesion and platelet aggregation [19], [20]. All these effects might be particularly relevant in ACS, a complex disease in which coronary vasoconstriction and inflammatory and thrombotic processes represent major pathogenetic factors.

In this study, we measured plasma concentration of metabolites involved in the NO biosynthetic pathway, in particular arginine, ADMA and SDMA, and we investigated their relationship with renal function and long-term outcome of patients with ACS.

## Materials and Methods

The Ethics Committee of the Centro Cardiologico Monzino approved the study, and all patients gave written, informed consent.

### Study population

This prospective study was conducted at the Centro Cardiologico Monzino, University of Milan. All consecutive patients who were admitted to the Coronary Care Unit (CCU) for non-ST-elevation acute myocardial infarction (NSTEMI) between September 1, 2005 and October 24, 2007 were enrolled in the study. We included patients who presented within 24 hours of the onset of symptoms (characteristic chest pain with electrocardiographic ST-segment depression or T wave inversion, and increase in troponin I). We excluded patients receiving long-term peritoneal or hemodialysis treatment. In order to prevent potential misclassification of patients who might have developed acute kidney injury, we also excluded patients with ACS-associated hemodynamic (acute pulmonary edema, cardiogenic shock) or electrical (life-threatening ventricular arrhythmias, high-degree conduction disturbances) instability or other major clinical complications at hospital presentation. Patients with angina precipitated by anemia or other correctable factors, severe valvular heart disease, malignancies, and severe liver disease, were also excluded.

### Study protocol

At hospital admission, before any pharmacologic therapy was started, venous blood samples were obtained for determination of arginine, ADMA and SDMA plasma concentrations. We also measured serum creatinine concentration in all patients at hospital admission. Glomerular filtration rate (eGFR) was estimated using the abbreviated Modification of Diet in Renal Disease (MDRD) equation [21]. Chronic kidney disease was categorized as an eGFR ≤60 ml/min per 1.73 m^2^ [22].

The choice of pharmacologic therapy and interventional strategy for management of ACS was left to the discretion of the CCU cardiologists. In all cases, early coronary angiography (within 24 hours from hospital presentation) was performed, unless contraindicated.

After hospital discharge, all patients were followed-up for at least one year. During the follow-up, a combined end-point of cardiac death and re-infarction (primary end point of the study) was considered.

Clinical results were analyzed according to the presence (CKD group) or absence (no-CKD group) of CKD.

For comparison, we considered a historical sample of 20 healthy subjects (mean age 60, range 56–69 years) and of 10 CKD patients (Stage 3 in all cases) without ACS (mean age 65, range 47–73 years; eGFR 51±7 ml/min per 1.73 m^2^) evaluated in our laboratory.

### Blood sampling and biochemical measurements

Blood was collected from the antecubital vein into EDTA containing tubes and plasma samples were obtained after centrifugation (3000 g, 10 min. at 4°C). Aliquots of plasma were stored at −80°C until analysis. Arginine, ADMA and SDMA plasma concentrations were measured by liquid chromatography method, performed using an HPLC system (ESA Bioscences, Chelmford, MA, USA) equipped with fluorimetric detector FP-1520 (Jasco, Tokyo, Japan) [23]. Briefly, organic solid phase extraction onto Oasis MCX SPE cartridge (Waters Milford, MA, USA) and basic derivatization with ortho-Phthaldialdehyde were previously performed after adding MMA (0.2 µM) as internal standard. Fifty µL of the purified sample were loaded onto Onyx Monolythic C18 HPLC column (100×4.6 mm, Phenomenex, Torrance, CA, USA) eluted at 2 mL/min by 7% acetonitrile in phosphate buffer 25 mM pH 6.5. Fluorescence was measured at excitation and emission wavelengths of 340 and 455 nm, respectively.

Data were obtained after comparison with calibration curves using arginine, ADMA and SDMA pure standard solutions. The intra- and inter-CVs % obtained with standard samples were <5% for all the analytics considered. The limit of detection of arginine was 0.041 µmol/L; it was 0.025 µmol/L for both methylarginines.

### Statistical analysis

Continuous variables are presented as mean±SD, and were compared using the *t*-test for independent samples. Variables not normally distributed are presented as median and interquartile ranges (IQR), and compared with the Wilcoxon rank-sum test. Categorical data were compared using the chi-square test or the Fisher exact test, as appropriate.

Pearson correlation analysis was used to examine the linear association between plasma arginine, ADMA, SDMA and eGFR.

Kaplan-Meier analysis was employed to generate time-to-event curves for the clinical end point (cardiac death and myocardial infarction), stratified according to values below or above medians of arginine, ADMA or SDMA. Multivariable-adjusted hazard ratios (HR) were computed by Cox proportional-hazard regression analysis.

All tests were two-tailed, and a P value of less than 0.05 was required for statistical significance. All calculations were computed with the aid of the SAS software package (Version 9.2 SAS Institute Inc, Cary, NC).

## Results

A total of 104 consecutive patients with NSTEMI were included in this study. Of these, 33 (32%) had CKD (Stage 3 in 29 patients and Stage 4 in 4 patients) and 71 (68%) had normal renal function. The baseline demographic and clinical characteristics of ACS patients with and without CKD are shown in Table 1. Patients with CKD were older and more likely to be treated with diuretics. Moreover, they had significantly lower hemoglobin levels and higher high-sensitivity C-reactive protein (hs-CRP) values.

**Table 1:** Baseline characteristics of the study patients.

	CKD group	no CKD group	P value
	(n = 33)	(n = 71)	
**Clinical characteristics**			
Age (yrs)	72±8	64±11	<0.001
Men, n (%)	22 (67%)	55 (77%)	0.24
Weight (kg)	74±13	76±13	0.46
Height (cm)	167±8	168±7	0.51
Smokers, n (%)	10 (30%)	24 (34%)	0.74
Diabetes mellitus, n (%)	11 (33%)	17 (24%)	0.31
Systemic hypertension, n (%)	19 (58%)	47 (66%)	0.39
Dyslipidemia, n (%)	19 (58%)	45 (63%)	0.57
Prior myocardial infarction, n (%)	8 (24%)	16 (23%)	0.84
Prior CABG, n (%)	5 (15%)	5 (7%)	0.28*
Prior PCI, n (%)	2 (6%)	7 (10%)	0.71*
Left ventricular ejection fraction, %	56±8	55±11	0.5
Medical therapy, n (%)	6 (18%)	8 (11%)	0.3
PCI, n (%)	22 (67%)	53 (75%)	0.39
CABG, n (%)	5 (15%)	10 (14%)	0.88
**Medications at hospital presentation**			
ACE inhibitor or ARB, n (%)	17 (52%)	30 (42%)	0.37
Aspirin, n (%)	23 (70%)	47 (66%)	0.72
Diuretics, n (%)	8 (24%)	7 (10%)	0.05
Statins, n (%)	12 (36%)	24 (34%)	0.79
Beta-blockers, n (%)	9 (27%)	19 (27%)	0.95
Calcium channel blockers, n (%)	8 (24%)	16 (23%)	0.84
Oral hypoglycemics, n (%)	10 (30%)	12 (17%)	0.12
**Laboratory measures**			
Serum creatinine (mg/dl)	1.37 (1.1–1.7)	0.98 (0.9–1.1)	NA
eGFR (ml/min/1.73 m^2^)	48±9	79±12	NA
Hemoglobin (g/dl)	13±1.5	14±1.5	0.002
Total cholesterol (mg/dL)	188±46	199±38	0.2
LDL cholesterol (mg/dL)	109±45	122±35	0.11
HDL cholesterol (mg/dL)	46±10	48±11	0.37
Triglycerides (mg/dL)	120 (88–184)	113 (88–185)	0.97§
hs-CRP (mg/L)	5.7 (4.4–8.7)	3.8 (2.2–5.8)	0.05§
Arginine (µmol/L)	62±17	68±18	0.14
ADMA (µmol/L)	0.46±0.1	0.42±0.1	0.35
SDMA (µmol/L)	0.65±0.2	0.42±0.1	<0.001

* By Fisher exact test.

§ by Wilcoxon Rank Sum Test.

ACE = angiotensin-converting enzyme; ARB = angiotensin II receptor blocker; CABG = coronary artery bypass graft surgery; CKD = chronic kidney disease; CRP = C-reactive protein; eGFR = estimated glomerular filtration rate; NA = not applicable; PCI = percutaneous coronary intervention.

**Figure 1:**
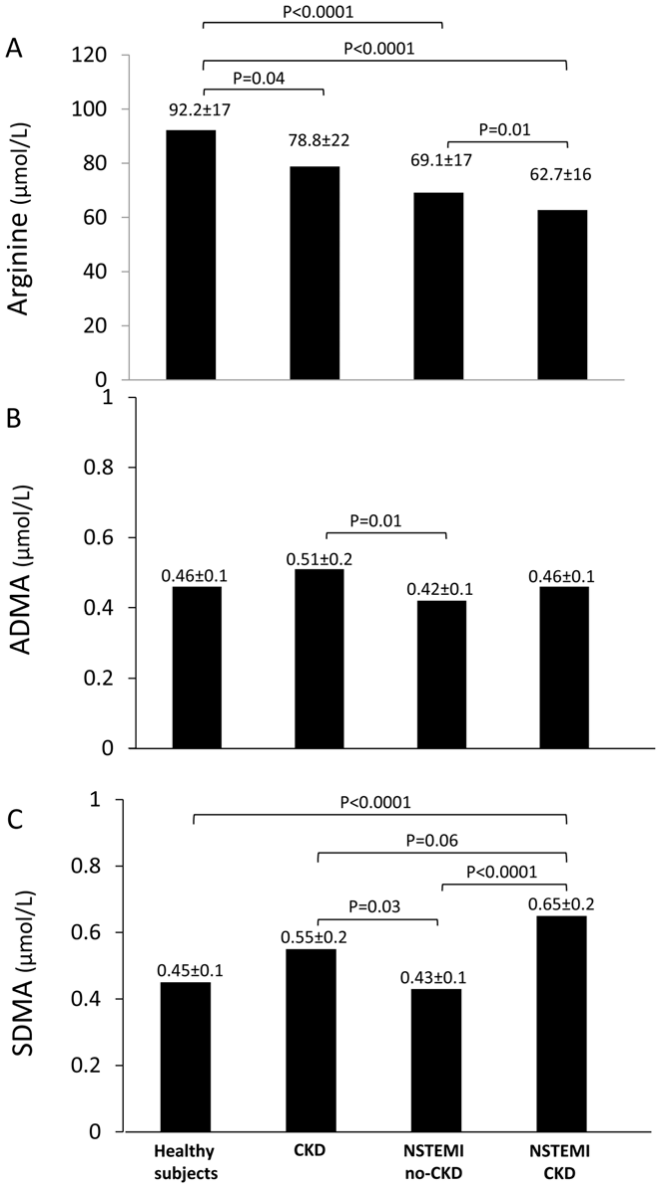
Arginine, ADMA and SDMA plasma levels. Arginine (panel A), asymmetric dimethylarginine (ADMA; panel B), and symmetric dimethylarginine (SDMA; panel C) plasma levels (mean±SD values) in healthy subjects (n = 20), in controls with chronic kidney disease (CKD; n = 10), and in NSTEMI patients without (n = 71) and with (n = 33) CKD.

In the whole population, plasma arginine was lower than our reference values in healthy subjects (67.1±16.7 µmol/L vs. 92.2±17.5 µmol/L, respectively; P<0.0001), whereas ADMA (0.435±0.09 µmol/L vs. 0.461±0.11 µmol/L, respectively; P = 0.25) and SDMA (0.501±0.19 µmol/L vs. 0.457±0.11 µmol/L, respectively; P = 0.35) were similar. Arginine and ADMA levels were not significantly different in CKD and no-CKD patients, whereas SDMA was significantly higher in CKD patients (Table 1). Figure 1 shows arginine, ADMA and SDMA values in healthy subjects, CKD controls, and in the two study groups. Arginine values were lower in CKD and ACS patients and ADMA levels were higher in CKD controls than in the other 3 groups. Finally, SDMA levels were higher in CKD patients than in patients with normal renal function.

**Figure 2:**
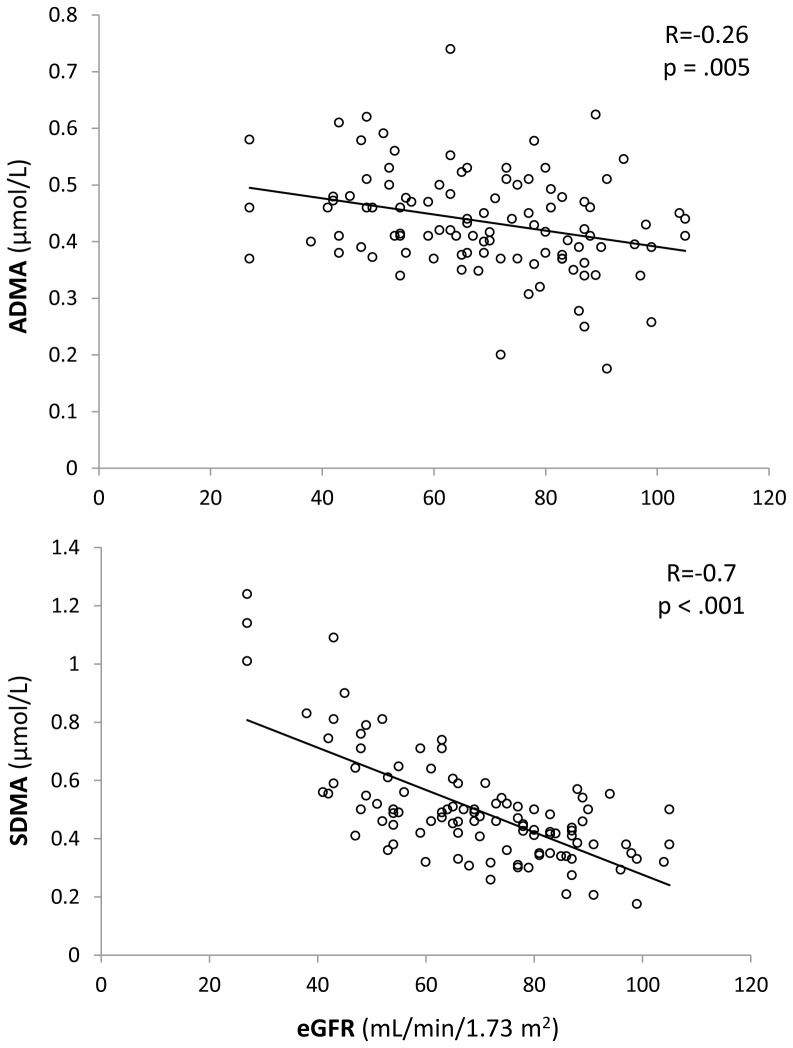
Relationships between NO synthesis inhibitors and renal function. Relationship between asymmetric (ADMA; upper panel) and symmetric (SDMA; lower panel) dimethylarginine plasma levels and estimated glomerular filtration rate (eGFR) in the study population.

Throughout the entire study population, both ADMA and SDMA were inversely correlated with eGFR, but this relationship was stronger for SDMA (Figure 2). No relationship, instead, was observed between arginine and eGFR (R = 0.14; P = 0.16). In addition, a significant correlation was observed between ADMA and SDMA levels (R = 0.52; P<0.0001) and between SDMA and hs-CRP values (R = 0.32; P = 0.018).

No patient died during hospital stay; 3 patients died during the follow-up (median 21 months; IQR 7–33): 2 (6%) patients in CKD group and 1 (1%) patient in no-CKD group. Overall, 24 (23%) patients reached the combined primary end-point of death and re-infarction during the follow-up: 12 (36%) patients in CKD group and 12 (17%) patients in no-CKD group (P = 0.02 in Kaplan-Meier analysis). Two (6%) and 14 (20%; P = 0.05) patients, respectively, underwent an elective percutaneous coronary procedure during the follow-up.

**Figure 3:**
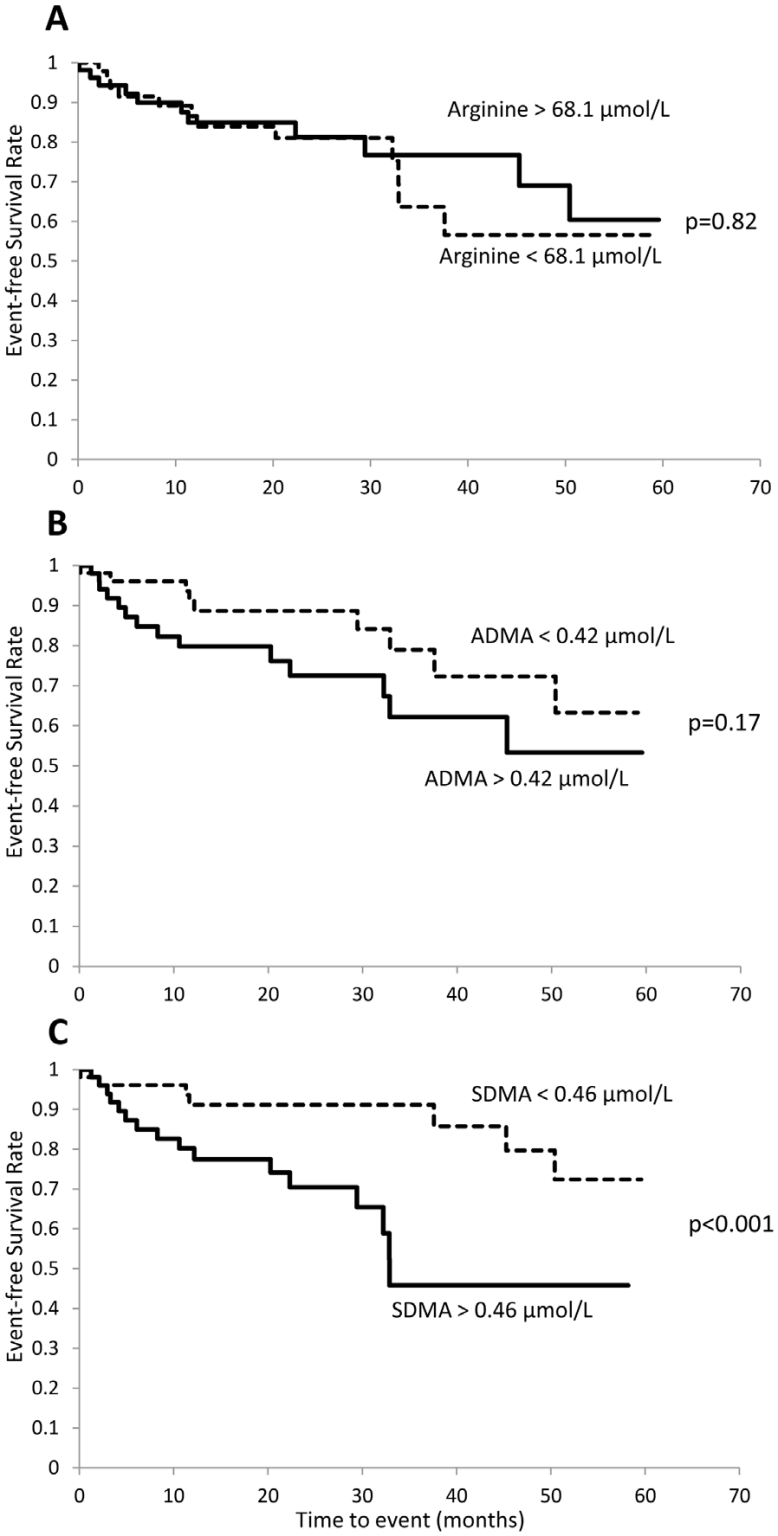
Kaplan-Meier survival analyses during follow-up. Composite outcomes of cardiac death and myocardial infarction according to concentrations of plasma arginine (panel A), asymmetric dimethylarginine (ADMA; panel B), and symmetric dimethylarginine (SDMA; panel C), divided by median levels. P values by log-rank test are shown.

Figure 3 shows the Kaplan-Meier curves according to median arginine (68.1 µmol/L), ADMA (0.42 µmol/L), and SDMA (0.46 µmol/L) values stratification in the study population. Notably, a significantly different incidence in the combined end point of death and re-infarction was observed only for patients stratified by median SDMA levels. Similar results were obtained when the Kaplan-Meier curves were analyzed comparing the upper tertile of arginine (>74.8 µmol/L; P = 0.33), ADMA (>0.47 µmol/L; P = 0.10) and SDMA (>0.5 µmol/L; P = 0.003) with the lower two tertiles.

Table 2 reports the HRs for the primary end point obtained by different Cox models. In models adjusted only for age, hemoglobin and left ventricular ejection fraction, HR for CKD and SDMA were high but, after mutual adjustment, only SDMA remained significantly associated with the primary end point.

**Table 2:** Cox regression analysis for the primary end point of the study (composite outcome of cardiac death and myocardial infarction).

Model	Variable	Adjusted HR	95% CI	P value
1	CKD	2.93	1.15–7.53	0.02
2	Arginine	0.95	0.37–2.42	0.91
3	ADMA	2.24	0.89–5.65	0.08
4	SDMA	6.80	2.09–22.2	0.001
5	Arginine	1.18	0.45–3.09	0.72
	CKD	3.04	1.16–7.97	0.02
6	ADMA	1.84	0.71–4.79	0.21
	CKD	2.52	0.96–6.64	0.06
7	SDMA	5.73	1.55–21.2	0.009
	CKD	1.35	0.49–3.71	0.55

Hazard ratios (HR) in models 1–4 are adjusted for age, hemoglobin and left ventricular ejection fraction; HRs in models 5–7 are also mutually adjusted.

HRs for CKD are vs. no-CKD; for all other variables, HRs are for values above vs. below median.

ADMA = asymmetric dimethylarginine; CKD = chronic kidney disease; CI = confidence intervals; SDMA = symmetric dimethylarginine.

## Discussion

The most significant finding of the present study is that plasma levels of SDMA, measured at hospital presentation, are elevated in NSTEMI patients with CKD, and are independent early predictors of the composite outcome of cardiac death and myocardial infarction.

Chronic kidney disease is present in a substantial proportion of patients with ACS and represents a potent and independent risk factor for short- and long-term adverse outcome [4]–[7], [9]. The reasons for this close association, however, are not completely clear and cannot be entirely explained by the clustering of the traditional cardiovascular risk factors. Besides clinical and therapeutic differences, it is likely that peculiar characteristics of renal insufficiency may play a considerable role [1], [24]. It has been hypothesized that the excessive risk associated with CKD can be attributed, at least in part, to endothelial dysfunction and reduced bioavailability of NO, a potential link between CKD and cardiovascular disease.

Elevated plasma concentrations of ADMA have been found in various clinical settings, ranging from critically ill patients admitted to the Intensive Care Unit [25] to patients with CKD [26] and end-stage renal disease [10], with stable coronary artery disease [27], and to those undergoing coronary angiography [28] and non-cardiac surgery [29]. In all these conditions, elevation of ADMA has been identified as an independent risk factor for future adverse cardiovascular events and death. It has been proposed that, by inhibiting NO synthesis, ADMA may contribute directly to endothelial dysfunction, depression of cardiac function, progression of chronic renal disease, and organ failure [12], [30], [31]. Recently, ADMA has also been measured in patients with ACS [32]–[34]. In a study by Cavusoglu et al. [33], plasma levels of ADMA were measured in a cohort of 182 men with ACS. The authors found that elevated plasma levels of ADMA were powerful and independent predictors of adverse cardiovascular outcomes. In particular, ACS patients who had the upper tertile of baseline ADMA had a significantly higher two-year mortality than those in the lower two tertiles combined (23.8% vs. 8.6%; P = 0.004).

In most previous studies, investigators focused mainly on ADMA and, until recently, little attention has been paid to the role of its structural isomer SDMA. To our knowledge, this is the first study evaluating the association between plasma levels of both dimethylarginines and long-term outcome in ACS. In our study, no increase in ADMA levels in plasma were found in comparison with healthy subjects, not even when the whole study group, as well as the two subsets of patients stratified according to the presence or absence of CKD, were considered. Moreover, no significant association between ADMA levels and clinical outcome was observed. Some important clinical (patients characteristics, type [STEMI vs. NSTEMI] and severity of ACS), methodological and analytical (ELISA vs. HPLC) differences among studies may explain the apparent inconsistency between our data and those previously published, regarding the capacity of ADMA to predict cardiac events in ACS [32]–[35].

Both dymethylarginines are physiologically present in plasma as a product of normal protein turnover. However, as SDMA is mainly eliminated by renal excretion, whereas ADMA is largely metabolized, it is not surprising to find a closer relationship between renal impairment and SDMA than with ADMA. The increase in SDMA observed in our study in CKD patients, as well as the good correlation found between SDMA and eGFR, are in agreement with a previous meta-analysis showing a strong correlation between SDMA and renal function [36]. As there is a high concentration of the ADMA degrading enzyme (DDAH) in the kidney, it is also conceivable that the decline in renal excretory function is paralleled by a reduction of DDAH activity (in the kidney). This, and the fact that about 10% of ADMA formed is also excreted by the kidneys, might explain why also ADMA is weakly related to parameters of renal function in some of the studies, including the present one.

When patients were stratified by median SDMA plasma concentration, a significant difference in long-term clinical outcome was found. Notably, SDMA was a stronger predictor of cardiac events, in particular re-infarction, than CKD, as defined according to GFR estimated by serum creatinine. The mechanism(s) linking SDMA and outcome, however, remains uncertain. Several mechanisms may potentially explain this association. First, SDMA might directly influence the outcome of ACS patients by participating to cause reduction in NO production and induction of endothelial dysfunction. Although SDMA has not been shown to directly affect NO synthase activity *in vitro* [37], we cannot exclude its influence on the production of NO in some clinical conditions characterized by its increase in plasma. Indeed, SDMA may have an indirect effect on NO synthesis, by inhibiting the y+ transporter that mediates the intracellular uptake of L-arginine and renal tubular arginine absorption [38], [39]. These two mechanisms might indirectly inhibit NO synthesis by interfering with L-arginine uptake. Second, SDMA accumulation in the plasma due to reduced renal clearance might only reflect kidney dysfunction. Many studies have shown a good correlation between SDMA and established estimates of GFR in humans, as well as in animal models [40]. Based on these premises, SDMA fulfills all criteria for an ideal GFR marker, i.e. stable production rate, free glomerular filtration, and lack of tubular reabsorption [36]. On the other hand, estimates of GFR from serum creatinine may lack in necessary sensitivity due to considerable inter-individual variability in muscle mass, protein intake, age, and sex [41]. This limitation is even more critical in ACS, a clinical setting in which no renal baseline conditions are available and serum creatinine levels at hospital admission cannot be considered a true stable value because the occurrence of a transient hemodynamic impairment, which in turn results in the increase of serum creatinine in the plasma. Moreover, creatinine increase may lag far behind glomerular filtration changes, because of its delayed rise after renal injury, due to the slow variations in its metabolism and the exponential relationship existing between these two variables. Thus, it is likely that SDMA is able to more accurately reflect glomerular filtration than creatinine and its levels in plasma may better identify the “true” CKD patients with a worse prognosis. According to this hypothesis, the lower SDMA values in CKD controls than in NSTEMI patients with CKD, might be explained by the higher eGFR in the former than in the latter group (51±7 and 48±9 ml/min per 1.73 m^2^, respectively). The list of mechanisms through which SDMA might directly contribute to unfavorable outcomes in ACS patients, however, is most likely not yet complete. In our study, SDMA levels in plasma were correlated with hs-CRP levels at hospital presentation, suggesting a possible role in promoting inflammatory response. A recent study has shown that SDMA is involved in the inflammatory process of CKD, by activating intracellular monocytic expression of interleukin 6 and tumor necrosis factor-alpha *in vitro*, whereas ADMA does not. This pro-inflammatory profile has been confirmed in a clinical study in which SDMA was associated with inflammatory markers [42]. Finally, a possible procoagulant state due to induction of tissue factor expression by peripheral monocytes, like that demonstrated for ADMA in ACS patients, cannot be excluded for SDMA [34].

Our data provide a basis for future studies which will investigate whether determination of SDMA, as well as its pharmacologic modulation, may help to guide care and improve outcomes of ACS patients. Irrespectively of its potential role in the pathophysiology of long-term adverse cardiac events, SDMA determination may have a considerable clinical usefulness in risk stratification of ACS patients. In particular, measurement of SDMA might allow for a more accurate and early recognition of CKD patients, who may require drug dose adjustments, renal prophylactic strategies, and targeted therapeutic interventions. Moreover, in these patients, long-term prognostic stratification may be more precise. Notably, in our study, we identified a potential cutoff value of SDMA of 0.46 µmol/L (median value) for risk classification.

Our study has some limitations. First, the size of the population is small and the findings need to be confirmed in larger studies. Similarly, the number of cardiac events is small, possibly due to the exclusion of high-risk patients from the study. Although this may represent an important limitation, the impossibility to differentiate between CKD and acute kidney injury on the bases of a single creatinine value measured at hospital admission, could have almost certainly lead to overestimate the number of patients with CKD. Nonetheless, the ADMA and SDMA levels in ACS patients developing acute kidney injury, as well as their possible association with short- and long-term outcomes, are not known and should be matter of future investigation. Second, being an observational study, our data do not unequivocally demonstrate that SDMA increase in plasma levels contributes to mortality and re-infarction. Further studies should investigate the relationship between SDMA increase and endothelial dysfunction, possibly evaluating endothelial-dependent vasodilatory response. Finally, we cannot exclude the possibility that some medications, taken by patients before hospital admission, may have influenced our results. Indeed, ADMA concentration has been shown to significantly decrease in ACS patients after a short-term medical therapy [32].

## Conclusions

In NSTEMI patients, CKD is associated with increased SDMA plasma levels and worse long-term prognosis. Further studies are needed to establish whether the increased mortality risk of CKD patients is due to SDMA-induced impaired NO synthesis and whether pharmacologic modulation of SDMA may improve their outcomes.
